# Vitamin E Biosynthesis and Its Regulation in Plants

**DOI:** 10.3390/antiox7010002

**Published:** 2017-12-25

**Authors:** Laurent Mène-Saffrané

**Affiliations:** Department of Biology, University of Fribourg, Chemin du Musée, 10, 1700 Fribourg, Switzerland; laurent.mene-saffrane@unifr.ch; Tel.: +41-268-008-808

**Keywords:** vitamin E, tocochromanol, tocopherol, tocotrienol, plastochromanol-8, tocomonoenol, homogentisate, polyprenyl pyrophosphate, nutrigenomics

## Abstract

Vitamin E is one of the 13 vitamins that are essential to animals that do not produce them. To date, six natural organic compounds belonging to the chemical family of tocochromanols—four tocopherols and two tocotrienols—have been demonstrated as exhibiting vitamin E activity in animals. Edible plant-derived products, notably seed oils, are the main sources of vitamin E in the human diet. Although this vitamin is readily available, independent nutritional surveys have shown that human populations do not consume enough vitamin E, and suffer from mild to severe deficiency. Tocochromanols are mostly produced by plants, algae, and some cyanobacteria. Tocochromanol metabolism has been mainly studied in higher plants that produce tocopherols, tocotrienols, plastochromanol-8, and tocomonoenols. In contrast to the tocochromanol biosynthetic pathways that are well characterized, our understanding of the physiological and molecular mechanisms regulating tocochromanol biosynthesis is in its infancy. Although it is known that tocochromanol biosynthesis is strongly conditioned by the availability in homogentisate and polyprenyl pyrophosphate, its polar and lipophilic biosynthetic precursors, respectively, the mechanisms regulating their biosyntheses are barely known. This review summarizes our current knowledge of tocochromanol biosynthesis in plants, and highlights future challenges regarding the understanding of its regulation.

## 1. Introduction

**Figure 1:**
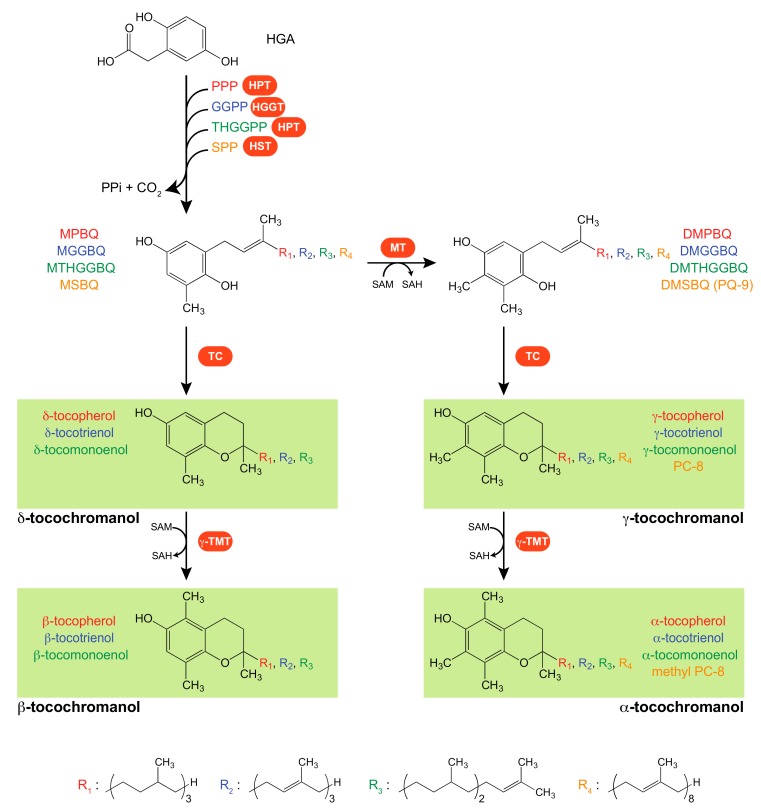
Tocochromanol biosynthetic pathways in plants. Tocochromanol and prenyl benzoquinol chemical structures and biosynthetic enzymes (highlighted in orange). Tocochromanol and prenyl benzoquinol names are color-coded to distinguish each tocochromanol pathway: red for the tocopherol pathway, blue for the tocotrienol pathway, green for the tocomonoenol pathway, and orange for the PC-8 and methyl PC-8 pathway. The α-, β-, γ-, and δ-forms of tocopherols, tocotrienols, and tocomonoenols have been identified in plants. For solanesyl-derived tocochromanols, only PC-8 has been identified in wild-type plants, and only methyl PC-8 has been identified in transgenic Arabidopsis overexpressing the γ-*TMT/VTE4* gene. Abbreviations: HGA, homogentisate; HGGT, homogentisate geranylgeranyltransferase; HPT, homogentisate phytyltransferase; HST, homogentisate solanesyltransferase; GGPP, geranylgeranyl pyrophosphate; γ-TMT, γ-tocopherol methyltransferase; MT, methyltransferase; PC-8, plastochromanol-8; PPi, pyrophosphate; PPP, phytyl pyrophosphate; PQ-9, plastoquinol-9; SAM, *S*-adenosyl-l-methionine; SAH, *S*-adenosyl-l-homocysteine; SPP, solanesyl pyrophosphate; TC, tocopherol cyclase; THGGPP, tetrahydrogeranylgeranyl pyrophosphate. Prenyl benzoquinol acronyms are detailed in the main text.

Natural compounds exhibiting vitamin E activity in animal cells belong to the chemical family of tocochromanols, a group of organic molecules with a polar chromanol ring and lipophilic polyprenyl side chain that varies according to the type of tocochromanol [1]. The polyprenyl precursor of tocopherols, tocotrienols, plastochromanol-8 (PC-8), and tocomonoenols is phytyl pyrophosphate (PPP), geranylgeranyl pyrophosphate (GGPP), solanesyl pyrophosphate (SPP), and tetrahydrogeranylgeranyl pyrophosphate (THGGPP), respectively (Figure 1). According to the degree of methylation of the chromanol ring, each type of tocochromanol exhibits up to four different forms, named α- (three-methyl groups), β- and γ- (two-methyl groups), and δ-tocochromanol (one-methyl group; Figure 1), respectively. While the four forms of tocopherol, tocotrienol, and tocomonoenol have been identified in wild-type plant extracts, only the γ- form of the solanesyl-derived tocochromanol PC-8 naturally exists.

The first article published on vitamin E relates the finding of an unknown nutritional factor that prevents embryo resorption during rodent gestation [2]. Several years later, this “*factor X*”, as it was originally named, was identified as α-tocopherol [3]. In addition to its essential role in animal reproduction, vitamin E has been shown to have beneficial roles in human health [4], notably by inhibiting lung cancer [5], by delaying brain aging and reducing the risk of developing Alzheimer’s disease [6], and by suppressing cholesterogenesis [7,8]. Although vitamin E is present in many edible plant-derived products, converging nutritional surveys conducted in both poor and developed countries, respectively, have shown that a significant proportion of human populations exhibit mild to severe vitamin E deficiencies [9,10,11,12,13]. It has been shown, for instance, that 23% of the Seoul metropolitan population exhibited plasma α-tocopherol concentrations below 12 μmol/L, the threshold defining vitamin E deficiency in humans [11]. The consequences of vitamin E deficiency on human health have not yet been fully documented nor investigated. However, it has been clearly established that low plasma vitamin E is strongly associated with miscarriage during the first trimester of woman pregnancy [12]. Moreover, it has been shown that diet supplementation with vitamin E decreased the miscarriage rate of pregnant woman by approximately 50% [12]. Collectively, these results demonstrate the importance of adequate vitamin E intake for proper reproduction.

It has been assumed for a long time that tocochromanol biosynthesis was the exclusive appanage of plants, algae, and some cyanobacteria that are all photosynthetic organisms. However, a recent study showed that *Plasmodium falciparum*, a non-photosynthetic parasite that causes malaria, synthesizes both α- and γ-tocopherols during its intraerythrocytic stages to avoid oxidative stress [14,15,16]. Besides this exception, tocochromanol metabolism has been primarily studied in angiosperms and in the vitamin E-producing cyanobacteria *Synechocystis*. The present review summarizes our current knowledge on tocochromanol metabolism in plants, including the core tocochromanol biosynthetic pathway that is now well delineated (Section 2); the transcriptional regulation of γ-*TMT* expression, which is the gene encoding the biosynthetic enzyme of the most potent vitamin E form of α-tocopherol in animals (Section 3); the regulation of homogentisate (HGA) biosynthesis, which is the polar precursor of tocochromanols (Section 4); and the regulation of polyprenyl pyrophosphate biosynthesis, which is the lipophilic precursor of tocochromanols (Section 5). Thus, this work complements the very recent review that highlighted the biosynthetic origins and transports of polar and lipophilic tocochromanol biosynthetic precursors in plants [17].

## 2. Tocochromanol Biosynthetic Pathways

Tocochromanol biosynthesis is initiated by the condensation of the polar aromatic head HGA with various lipophilic polyprenyl pyrophosphates that determine the type of tocochromanol. PPP is the lipophilic biosynthetic precursor of tocopherols, while GGPP is the one for tocotrienols, as is SPP for PC-8, and THGGPP for tocomonoenols (Pellaud and Mène-Saffrané, 2017 [17] and Figure 1). The condensation reaction is catalyzed by three types of HGA prenyltransferases that possess each their substrate specificities. Tocopherol synthesis is initiated by HGA phytyltransferases (HPTs) that condense HGA and PPP. This enzyme has been identified in both Arabidopsis and the cyanobacterium *Synechocystis* [18,19]. While the Arabidopsis HPT (AtHPT), also named VTE2, preferentially utilizes PPP as a prenyl donor, its *Synechocystis* counterpart uses both PPP and GGPP [18]. Although AtHPT poorly utilizes GGPP as a substrate in vitro, and wild-type Arabidopsis accessions do not naturally accumulate tocotrienols, it has been shown that AtHPT catalyzes the synthesis of tocotrienols in transgenic Arabidopsis plants that are overaccumulating HGA [20]. Recently, AtHPT was also shown to be catalyzing the accumulation of γ-tocomonoenol in Arabidopsis seeds, indicating that THGGPP is a substrate for this enzyme [21]. Collectively, these data show that the substrate specificity of HGA phytyl transferases is wider than that originally described, and is notably modulated by HGA availability. In Poaceae, tocotrienol synthesis is initiated by HGA geranylgeranyltransferase (HGGT), which utilizes both GGPP and PPP as prenyl donors in vitro. In addition, overexpression of the barley *HGGT* gene in the Arabidopsis *vte2* mutant induces the quantitative accumulation of both tocotrienols and tocopherols in seeds and leaves, indicating that HGGT utilizes both prenyl donors in vivo as well [22]. The Arabidopsis *vte2-1* mutant lacks tocopherols, but still accumulates PC-8 in both seeds and leaves, confirming in vitro data showing that VTE2 is not involved in the condensation of HGA with the solanesyl derivative SPP [18,23]. This reaction is catalyzed by the HGA solanesyltransferase (HST) that has been identified in both *Chlamydomonas* and Arabidopsis [24,25]. The condensation reaction between HGA and polyprenyl pyrophosphates produces 2-methyl-6-phytyl-1,4-benzoquinol (MPBQ) for tocopherols, 2-methyl-6-geranylgeranyl-1,4-benzoquinol (MGGBQ) for tocotrienols, 2-methyl-6-solanesyl-1,4-benzoquinol (MSBQ) for PC-8, and 2-methyl-6-tetrahydrogeranylgeranyl-1,4-benzoquinol (MTHGGBQ) for tocomonoenols (Figure 1). These compounds are either direct precursors of δ- and β-tocochromanols, or can alternatively be methylated by a methyltransferase (MT/VTE3) that uses *S*-adenosyl-l-methionine (SAM) as a methyl donor [26,27]. The products of the later reaction are prenylated dimethyl-benzoquinols, namely 2,3-dimethyl-6-phytyl-1,4-benzoquinol (DMPBQ) for tocopherols, 2,3-dimethyl-6-geranylgeranyl-1,4-benzoquinol (DMGGBQ) for tocotrienols, 2,3-dimethyl-6-solanesyl-1,4-benzoquinol better known as plastoquinol-9 (PQ-9) for PC-8, and 2,3-dimethyl-6-tetrahydrogeranylgeranyl-1,4-benzoquinol (DMTHGGBQ) for tocomonoenols (Figure 1). Both methyl- and dimethyl-benzoquinols are then further cyclized by the tocopherol cyclase (TC/VTE1) into δ- and γ-tocochromanols, respectively. Seeds of the Arabidopsis *vte1-1* mutant lack all tocopherols, PC-8, and γ-tocomonoenol, indicating that the cyclase indiscriminately uses mono- and dimethyl-benzoquinols carrying either a phytyl, a solanesyl, or a tetrahydrogeranylgeranyl side chain as substrates [21,23,28,29]. The fourth and final step of tocochromanol biosynthesis consists of the methylation of γ- and δ-tocochromanols into α- and β-tocochromanols, respectively [30,31]. This reaction is catalyzed by the γ-tocopherol methyltransferase (γ-TMT/VTE4) that utilizes SAM as methyl donor (Figure 1). In Arabidopsis leaves and seeds, VTE4 converts γ- and δ-tocopherols into α- and β-tocopherol, respectively, indicating that the methyl transferase efficiently methylates phytyl-derived tocochromanols [30]. In addition, transgenic Arabidopsis lines overexpressing the barley *HGGT* gene notably produce α-tocotrienol [32]. This indicates that the γ-tocopherol methyltransferase VTE4 catalyzes that methylation of geranylgeranyl-derived tocochromanol such as γ-tocotrienol as well.

## 3. Regulation of the γ-*tocopherol methyltransferase* Expression

The different tocochromanol forms identified in plants do not exhibit the same vitamin E activity in animal cells [1]. Based on the rat fetal resorption assay, α-tocopherol (1.49 IU/mg; 100%) is by far the most potent vitamin E form, followed by β-tocopherol (0.75 IU/mg; 50%), α-tocotrienol (0.45–0.75 IU/mg; 30–50%), γ-tocopherol (0.15 IU/mg; 10%), β-tocotrienol (0.08 IU/mg; 5%), and δ-tocopherol (0.05 IU/mg; 3%). The vitamin E activities of γ- and δ-tocotrienol have been tested and were below the limit of detection, while those of other tocochromanols such as PC-8 and tocomonoenols have not been tested yet. The difference in vitamin E activity among tocochromanol forms results from their differential affinities to the α-tocopherol transfer protein, a cytoplasmic protein that transports tocochromanols from the endosomal fraction of hepatocytes into the bloodstream of animals [33,34]. This implies that at equal molarity, a plant tissue accumulating α-tocopherol instead of γ-tocopherol for instance, exhibits 10 times more vitamin E activity. Based on this, a relevant question regarding the regulation of tocochromanol biosynthesis in plants and the improvement of crops vitamin E activity is: what are the molecular mechanisms regulating γ-*TMT* expression?

The main tocochromanol identified in wild-type leaves analyzed to date is α-tocopherol, indicating that leaf γ-TMT/VTE4 activity is sufficient to quantitatively methylate the γ-tocopherol pool present in this tissue. In contrast, despite the presence of PC-8 (γ-tocochromanol) in wild-type Arabidopsis and maize leaves, methyl PC-8 (α-tocochromanol) has not been detected in any wild-type tissue analyzed to date [23]. In contrast, methyl PC-8 accumulates in transgenic Arabidopsis leaves overexpressing γ-*TMT/VTE4,* demonstrating that PC-8 is a substrate for γ-TMT/VTE4, and that endogenous γ-TMT/VTE4 activity is not sufficient to convert all γ-tocochromanols into α-tocochromanols [35]. This later conclusion is further supported by the tocochromanol composition of transgenic Arabidopsis leaves overexpressing the barley *HGGT* gene. Indeed, while these transgenic lines accumulate tocopherols mostly under the form of α-tocopherol, their tocotrienol pool is mainly under the form of γ-tocotrienol [32]. In contrast to this, another metabolic engineering study in which tocochromanol biosynthesis has been increased by enhancing the production of HPP in chloroplasts suggests that γ-TMT activity might be limiting for α-tocopherol synthesis in leaves. In comparison to controls, these Arabidopsis transgenic lines overaccumulated both γ-tocopherol and γ-tocotrienol, but exhibited control levels for α-tocopherol in leaves [36]. In addition, they did not accumulate any detectable α-tocotrienol, despite the strong accumulation of γ-tocotrienol in leaf tissues. In alfalfa, it has been shown that the overexpression of a Medicago γ-*TMT* modestly but significantly increased α-tocopherol accumulation in leaves [37]. Collectively, these data show that the leaf γ-TMT/VTE4 activity is clearly not sufficient to fully methylate both γ-tocotrienol and PC-8, and is sometimes limiting α-tocopherol biosynthesis. The former conclusion might indicate that γ-TMT/VTE4 has a lower affinity for γ-tocotrienol and PC-8 than for α-tocopherol.

In contrast to leaves, the tocochromanol composition of wild-type seeds is much more variable, and three groups of plant species can be currently distinguished according to this criterion. The seeds of some plant species accumulate mostly γ-tocochromanols and very little or no α-tocochromanols. This is notably the case of wild-type Arabidopsis seeds, which accumulate mostly γ-tocopherol and PC-8, very low levels of α-tocopherol, and no methyl PC-8 [23]. The overexpression of γ-*TMT* genes in Arabidopsis and *Brassica napus* is sufficient to trigger the quantitative accumulation of both α-tocopherol and methyl PC-8 in transgenic seeds, thus demonstrating that the lack of α-tocochromanols in the seeds of these species results from the very low transcriptional activity of the γ-*TMT/VTE4* gene in seeds [30,35,38]. In contrast to Arabidopsis, the seeds of other plant species mostly accumulate α-tocochromanols, and very few γ-tocochromanols. This is notably the case of sunflower and wheat, which accumulate mostly α-tocopherol ± α-tocotrienol, and very low levels of γ-tocochromanols [39,40]. This indicates that the VTE4 activity in seeds of these species is sufficient to quantitatively convert almost all of the γ-tocochromanols into α-tocochromanols. The third type of plants accumulate both α- and γ-tocochromanols in seeds, suggesting that although their VTE4 activity is sufficient to support the synthesis of α-tocochromanols, it is not strong enough to fully methylate the pools of γ-tocochromanols. This group includes species such as rapeseed and maize [41,42,43]. Our current knowledge of the transcriptional mechanism(s) regulating γ-*TMT/VTE4* expression, notably in seeds, is currently limited to a single study performed in soybean. Tocopherol analysis in seeds of 1109 wild and cultivated soybean varieties showed that while almost all of them produce primarily γ-tocopherol, three varieties accumulate up to seven times more α-tocopherol [44]. Quantitative trait loci (QTL) analysis in high α-tocopherol soybean varieties showed that polymorphism located in the γ-*TMT3* promoter correlated with the higher expression of the methyl transferase, and thus that α-tocopherol level is transcriptionally regulated in soybean seeds as well [45]. Collectively, these data demonstrate that the synthesis of α-tocochromanols, such as α-tocopherol in seeds and leaves, is determined by the mechanism(s) regulating the expression of the γ-*TMT/VTE4* gene. To date, despite many independent research initiatives that led to the identification of numerous α-tocopherol QTLs in Arabidopsis [46], soybean [45,47,48], maize [42,43,49,50], winter oilseed rape [51,52], and barley [53], the transcriptional machinery regulating the expression of γ-*TMT* genes in plants is still unknown.

## 4. Plastidic Availability in Homogentisate Regulates Tocochromanol Synthesis

**Figure 2:**
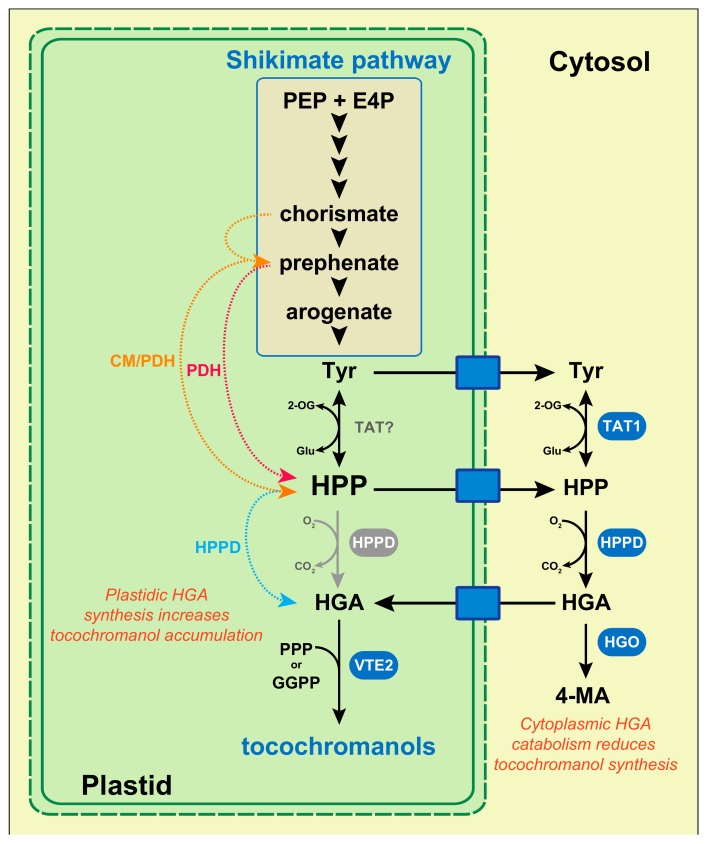
Biosynthesis and transport of homogentisate in plants. Red, orange, and blue dotted lines correspond to transgenic plants overexpressing the coding sequence of yeast *PDH*, bacterial *CM/PDH*, and plant *HPPD,* respectively, all fused to a sequence encoding a chloroplast transit peptide. Biosynthetic enzymes demonstrated to be involved in tocochromanol synthesis are highlighted in blue or gray. In species such as Arabidopsis, HPPD is localized in the cytoplasm. In contrast, in maize, tomato, and cotton, *HPPD* genes exhibit a typical chloroplast transit signal, suggesting that this enzyme is localized in the chloroplasts of these species (HPPD in grey). In soybean, HPPD have been localized in both compartments. Plant species in which HGA biosynthesis is localized in the cytosol must have chloroplast membrane transporters (blue boxes) exporting Tyr and HPP into the cytosol, and importing HGA back into chloroplasts. Abbreviations: CM/PDH, bacterial bi-functional chorismate mutase/prephenate dehydrogenase; E4P, erythrose 4-phosphate; GGPP, geranylgeranyl pyrophosphate; Glu, glutamate; HGA, homogentisate; HGO, homogentisate dioxygenase; HPP, 4-hydroxyphenylpyruvate; HPPD, 4-hydroxyphenylpyruvate dioxygenase; 4-MA, 4-maleylacetoacetate; 2-OG, 2-oxoglutarate; PDH, prephenate dehydrogenase; PEP, phosphoenolpyruvate; PPP, phytyl pyrophosphate; TAT, tyrosine aminotransferase; Tyr, l-tyrosine.

Early feeding experiments of safflower, sunflower, and soybean cell cultures with HGA have shown that an exogenous HGA supply significantly increased tocochromanol biosynthesis in plant cells [54,55,56]. These indicate that the endogenous mechanism(s) regulating HGA biosynthesis and availability in plant cells directly determines the final amount of tocochromanols. The tocochromanol biosynthetic precursor HGA comes from the degradation of the aromatic amino acid l-tyrosine (l-tyr), which is produced by the plastidic shikimate pathway (Figure 2). l-tyr is first converted into 4-hydroxyphenylpyruvate (HPP) by tyrosine aminotransferases (TATs). Among the six to 10 *TAT* genes identified in the genetic model Arabidopsis, the enzymatic activity has been experimentally confirmed only for TAT1 (also named TAT7) and TAT2 [57,58,59,60]. Regarding tocochromanol synthesis, it has been shown that TAT1/TAT7 controls 35–50% of α-tocopherol biosynthesis in Arabidopsis leaves [59]. The other TAT(s) involved in the TAT1-independent tocochromanol biosynthesis, as well as the ones involved in seed tocochromanol biosynthesis, have not been identified yet. Following tyrosine transamination, HPP is converted by 4-hydroxyphenylpyruvate dioxygenase (HPPD) into HGA (Figure 2). A corpus of biochemical, genetic, and cell biology data indicate that the cellular compartment hosting HGA biosynthesis is different among plant species. This aspect of tocochromanol biosynthesis has been recently reviewed in detail [17]. In the genetic model Arabidopsis, several studies have provided evidence that HGA biosynthesis is confined to the cytoplasm. The sequence analysis of the Arabidopsis *TAT1/TAT7* and *HPPD* genes have shown that both lack the typical sequence encoding for a chloroplast transit peptide, suggesting that TAT1/AtTAT7 and HPPD are localized in the cytoplasm [60]. This has been confirmed by Western blot for HPPD, and with TAT1:GFP and HPPD:GFP fusion proteins, which were both localized in the cytoplasm of Arabidopsis cells [60,61].

In addition, *HPPD* genes isolated from barley, carrot, and coleus also lack chloroplast transit peptide sequences [62]. Collectively, these demonstrate that HGA biosynthesis, at least in these species, is exclusively localized in the cytoplasm. This data has major consequences regarding the regulation of tocochromanol biosynthesis in these species, since the cytoplasmic HGA biosynthesis implies the existence of chloroplast membrane transporters that export l-tyr/HPP into the cytoplasm, and other one(s) that import HGA back into chloroplasts (Figure 2). Thus, beyond HGA biosynthesis *per se*, these chloroplast membrane transporters might constitute additional levels of regulation of plastidic HGA availability, and thus determine the final amount of tocochromanol produced by a given tissue.

Genetic data obtained from soybean further illustrate how the cytoplasmic HGA biosynthesis and the exchanges of tocochromanol biosynthetic precursors between chloroplasts and the cytoplasm participate in the regulation of tocochromanol biosynthesis in plants. In this species, the HPPD activity is encoded by a single gene that exhibits two transcription start sites. It has been shown that the long transcript encodes a polypeptide imported into the chloroplast, while the short one encodes a polypeptide that remains in the cytoplasm [63]. This indicates that soybean HPPD activity is localized in both the cytoplasm and chloroplasts. In addition, seeds of the soybean *hgo1* mutant, which carries a mutated *HOMOGENTISATE DIOXYGENASE 1* gene encoding an enzyme that catabolizes HGA into 4-maleylacetoacetate (Figure 2), exhibits higher amounts of both HGA and tocochromanols [64]. Interestingly, the HGO1 activity catabolizing HGA is localized in the cytoplasm of soybean cells, indicating that the cytoplasmic HGA catabolism negatively impacts tocochromanol biosynthesis in this species. Similarly, the Arabidopsis HGO gene (At5g54080) encodes a protein expected to be localized in the cytoplasm, according to the The Arabidopsis Information Resource website. Collectively, these data demonstrate that the cytoplasmic HGA synthesis and catabolism in species such as Arabidopsis and soybean are two mechanisms that determine the final amount of tocochromanols in seeds. Moreover, they reveal that yet-unknown chloroplast membrane transporters exporting l-tyr/HPP into the cytoplasm, and other one(s) importing HGA back into chloroplasts, are potentially limiting factors for tocochromanol accumulation. To date, neither the membrane chloroplast transporters exporting l-tyr and HPP in the cytoplasm, nor the one importing HGA back into chloroplasts, have been identified in Arabidopsis. In contrast, a cationic amino acid transporter localized in the chloroplast membrane and capable of exporting phenylalanine, tryptophan, and l-tyr from the stroma into the cytosol has been identified in petunia [65].

The major role of plastidic HGA availability and its transport from the cytoplasm into plastids in the regulation of tocochromanol biosynthesis can be deduced from four metabolic engineering studies that aimed at increasing plant vitamin E activity. In higher plants, HPP derives from the sequential catabolism of l-tyr into chorismate, prephenate, and arogenate (Figure 2). In plants, l-tyr restricts its own biosynthesis, and thus the one of tocochromanols, by feedback inhibition of both arogenate dehydrogenase and prephenate dehydrogenase [20]. In contrast, prokaryotes and yeast produce HPP directly from chorismate via bi-functional chorismate mutase (CM)/prephenate dehydrogenase (PDH) activity, and from prephenate via PDH activity, respectively (Figure 2). It has been previously shown that the overexpression of bacterial *CM/PDH* or yeast *PDH* genes in transgenic plants, including Arabidopsis, strongly increases the accumulation of both HGA and tocochromanols [20,56,66]. Interestingly, both *CM/PDH* and *PDH* coding sequences were fused to a sequence encoding a chloroplast transit peptide and expressed in combination with *HPPD* genes that were also fused to a sequence encoding a chloroplast transit peptide. Collectively, these studies demonstrate that bypassing the plant l-tyr feedback inhibition and functionalizing chloroplasts with the HPPD activity significantly stimulate tocochromanol metabolism in plants.

Interestingly, the overexpression of bacterial *CM/PDH* or yeast *PDH* genes alone is more effective in terms of tocochromanol biosynthesis in Arabidopsis leaves than when these genes are co-expressed in combination with *HPPD* [20,36]. Compared with transgenic Arabidopsis overexpressing *CM/PDH* alone, the lower tocochromanol accumulation in transgenic Arabidopsis leaves carrying both transgenes (*CM/PDH* or *PDH* with *HPPD*) correlates with a much higher accumulation of free HGA [20]. This indicates that whereas HGA availability is limiting for tocochromanol accumulation, a high HGA concentration likely inhibits their biosyntheses. In addition to revealing the potentially toxic effect of HGA on tocochromanol metabolism, these two later studies indicate that the HPPD activity and HPP/HGA trafficking across chloroplast membranes are both not limiting in Arabidopsis leaves. Indeed, the overaccumulation of tocochromanols resulting from the sole overexpression of *CM/PDH* or *PDH* in chloroplasts implies that HPP is efficiently exported into the cytosol where the HPPD activity is localized, and that the cytoplasmic HGA is imported back into chloroplasts to sustain tocochromanol biosynthesis (Figure 2). Since tocochromanol biosynthesis is strongly increased by the overexpression of a bacterial *CM/PDH* gene alone in Arabidopsis leaves, one can conclude that the endogenous HPPD activity (cytoplasmic) and HPP/HGA trafficking are both not so limiting for leaf tocochromanol metabolism in Arabidopsis. In contrast to this, the situation seems very different in tobacco leaves; while the overexpression of the yeast *PDH* gene does not significantly alter tocochromanol accumulation in transgenic tobacco leaves, its overexpression in combination with *HPPD* fused to a chloroplast targeting sequence strongly increases tocochromanol accumulation [66]. The reasons explaining these differences between Arabidopsis and tobacco leaves are currently unknown but suggest that HPPD activity and/or HPP/HGA trafficking in tobacco is limiting compared to Arabidopsis leaves.

**Figure 3:**
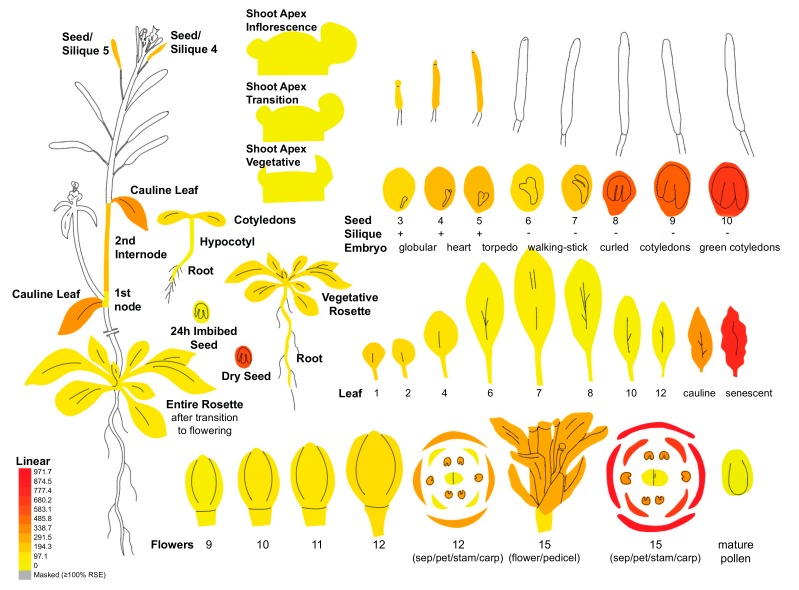
*HPPD* expression pattern during Arabidopsis development. The expression of the Arabidopsis *HPPD* gene (At1g06570) was assessed with ePlant (http://bar.utoronto.ca/eplant). *HPPD* expression level is equal to 697.5 in green cotyledons (SD = 36.3; *n* = 3), and is the third highest *HPPD* expression after sepals (level = 971.7; SD = 84.2; *n* = 3) and senescent leaves (level = 797.7; SD = 6.9; *n* = 3).

In Arabidopsis seeds, re-analysis of previously published data indicates that HPP/HGA trafficking across the chloroplast membranes is much less efficient than in leaves despite the significant tocochromanol metabolism in this tissue. Co-expression of *CM/PDH* in combination with *HPPD* in Arabidopsis stimulates seed tocochromanol accumulation, whereas the expression of *CM/PDH* alone does not significantly alter it (construct pMON36596 versus pMON36520 in the Supplementary data of Karunanandaa et al. [56], 2005, respectively). In this study, a sequence encoding a chloroplast transit peptide was fused to both *CM/PDH* and *HPPD* sequences as well, showing that seed tocochromanol might be enhanced in seeds if seed chloroplasts are functionalized with both activities. This indicates that the endogenous HPPD activity and/or HPP/HGA trafficking is much less efficient in Arabidopsis seeds than in leaves. However, the *HPPD* gene is strongly expressed in Arabidopsis seeds, a tissues in which *HPPD* expression is among its highest during plant development (Figure 3).

This indicates that HPPD activity is likely not limiting tocochromanol metabolism in Arabidopsis seeds, and that in contrast, HPP/HGA trafficking restricts tocochromanol biosynthesis in these tissues. This novel interpretation of old data also suggests that HPP/HGA trafficking across the chloroplast membrane is likely a target of choice to increase tocochromanol biosynthesis in seeds.

The tocochromanol patterns of transgenic plants expressing bacterial *CM/PDH* and yeast *PDH* alone or in combination with *HPPD* vary according to the plant species, organ, and developmental stage. Thus, while young tobacco and Arabidopsis leaves overexpressing *CM/PDH* or *PDH* genes overaccumulate primarily tocotrienols, mature Arabidopsis transgenic leaves expressing these constructs accumulate mostly tocopherols [20,36,66]. In contrast to leaves, Arabidopsis and soybean transgenic seeds expressing *CM/PDH* or *PDH* genes primarily overaccumulate tocotrienols [56]. Since the type of tocochromanol results from the polyprenyl pyrophosphate substrate, these results indicate that the tocopherol precursor PPP is likely limiting in seeds and young leaf tissues, whereas GGPP is not.

## 5. Regulation of Polyprenyl Pyrophosphate Availability

Since the type of tocochromanol (tocopherol, tocotrienol, PC-8, tocomonoenol) is determined by the polyprenyl pyrophosphate substrate condensed to HGA, the biosynthetic pathways producing the lipophilic tocochromanol precursors (PPP, GGPP, SPP, and THGGPP) are key components in the regulation of tocochromanol biosynthesis. To date, although the tocotrienol biosynthetic pathway is well characterized, and key tocotrienol biosynthetic genes have been identified in plants, there are currently no data describing the mechanisms regulating the biosynthesis of GGPP used for tocotrienol biosynthesis [32]. This may result from the fact that the wild accessions of the genetic model Arabidopsis do not accumulate the tocotrienols that are mostly prevalent in the seeds of Poaceae. As the GGPP that is used for tocotrienol synthesis likely originates from the plastidic methyl erythritol phosphate pathway (MEP), one can assume that the regulation of tocotrienol biosynthesis might be directly linked to this pathway. In addition, the mapping of the tocotrienol QTLs identified in maize and barley grains might improve our understanding of the mechanisms regulating its accumulation [67,68]. It is known that tocotrienols accumulate in plants or tissues that do not usually produce them when HGA is overaccumulated, such as in transgenic Arabidopsis plants expressing *CM/PDH* or *PDH* genes, or in the leaves of transgenic tobacco carrying similar constructs [20,36,56,66]. The current interpretation of these data is that GGPP is likely not limiting in these species or tissues, and that HGA overaccumulation alters the specificity of the tocopherol prenyltransferase. These results might indicate that HGA availability in Poaceae seeds might contribute to the regulation of tocotrienol biosynthesis in these species.

The biosynthesis of PC-8 and its solanesyl benzoquinone precursor plastoquinone-9 (PQ-9) is initiated by the homogentisate solanesyltransferase-dependent condensation of HGA with SPP that comes from the preferential concatenation of GGPP and isopentenyl pyrophosphate [25,69,70,71]. To date, four genes have been found to specifically regulate PQ-9/PC-8 accumulation in plants. The *Nicotinamide Adenine Dinucleotide Phosphate* (*NADPH*) *dehydrogenase C1* (*NDC1*; AT5G08740) is a type II NAD(P)H quinone oxidoreductase that reduces PQ-9 into PQH_2_-9, using ferredoxin as an electron donor [72]. This enzyme is localized in plastoglobuli together with prenyl quinones/quinols and tocochromanols such as PC-8. In Arabidopsis *ndc1* mutants, the leaf PQ pool is more oxidized than in wild-type controls, while PC-8 amounts are reduced by ca 66% [72]. In contrast, leaf tocopherol levels were not affected by the mutation. The proposed model explaining the role of NDC1 in PC-8 accumulation is based on tocopherol cyclase—the enzyme that notably catalyzes the cyclization of PQH_2_-9 into PC-8—preferentially using reduced prenyl quinones [73]. Two ABC1 atypical kinases, *ABC1K1* (AT4G31390) and *K3* (AT1G79600), have been shown to regulate PQ-9/PC-8 metabolism in Arabidopsis [74,75,76]. Both kinases are abundant in plastoglobuli, and single and double *abc1k1* and *abc1k3* mutants exhibit altered PQ-9/PC-8 metabolism. Although the function of these atypical kinases in PQ-9/PC-8 metabolism has been clearly demonstrated, their mode(s) of action remains to be determined. It has been shown that both ABC1K1 and K3 phosphorylate the tocopherol cyclase VTE1. Since the biosynthesis of tocopherols, which requires VTE1 activity as well, was not affected in *abc1k3* leaves, it is unlikely that the ABC1K3 mode of action is mediated by VTE1 phosphorylation. In contrast, tocopherol amounts were significantly reduced in *abc1k1* leaves, suggesting that the ABC1K1 mode of action might involve the regulation of the tocopherol cyclase activity. The role of ABC1K1 and K3 in tocochromanol metabolism might also be mediated by the synthesis of fribrillin, a group of lipid-associated proteins that are localized in chloroplasts and essential for the biosynthesis of PQ-9, and thus PC-8 [77,78]. Indeed, Arabidopsis *fibrillin-5* mutants exhibit lower PQ-9/PC-8 amounts, and it was shown that mutation in *abc1k3* strongly reduces the accumulation of fibrillin proteins [74,77].

Regarding the regulation of tocopherol biosynthesis, it is known that the availability of phytol is a major parameter governing the final amount of tocopherols in a plant tissue. It has been shown, for instance, that the supplementation of plant cell suspensions with free phytol is sufficient to significantly increase tocopherol accumulation in these cells [54,55,56]. Moreover, feeding Arabidopsis seedlings with radiolabeled phytol has been associated with the biosynthesis of radiolabeled α-tocopherol, thus suggesting that free phytol is likely recycled into tocopherols [79]. The first genetic evidence supporting this biosynthetic model was provided by the Arabidopsis *vte5* that lacks 80% and 65% of the tocopherols in seeds and leaves, respectively [80]. Recently, the VTE5-dependent biosynthesis of tocopherols has also been reported in tomato leaves and fruits [81]. VTE5 is a phytol kinase that phosphorylates the free phytol that is released during chlorophyll catabolism into phytyl phosphate [80]. The chlorophyll-dependent tocopherol biosynthesis is further supported by genetic data provided by the Arabidopsis *g4/chlsyn1* mutant, which carries a mutated chlorophyll synthase that esterifies geranylgeranyl pyrophosphate on chlorophyllide *a* [82]. The leaves of this mutant grown in vitro on a media supplemented with sucrose are albino, and do not accumulate tocopherols. In addition, *g4/chlsyn1* seeds lack 75% of the tocopherols, thus indicating that tocopherol biosynthesis is also mostly chlorophyll-dependent in Arabidopsis seeds as well. Collectively, these data demonstrate that the PPP used for tocopherol biosynthesis primarily originates from the recycling of phytol released during chlorophyll catabolism in both leaves and seeds. This biosynthetic model, including details about enzymes such as Mg-dechelatase, chlorophyllases, and pheophytinase, has been very recently reviewed, and will not be developed here [17].

If one can easily understand the origin of PPP in the seeds of chloroembryophytes such Arabidopsis or rapeseed, which accumulate significant amounts of chlorophylls in developing embryos, how do leucoembryophyte seeds produce tocopherols, since they *a priori* do not synthetize chlorophylls? A comprehensive analysis of vitamin E natural variation in maize grain recently provided an unexpected answer to this puzzling question. Among the 52 QTLs identified for maize grain tocochromanols, QTL5 and QTL24 exhibited scores for phenotypic variance explained among the highest of all identified QTLs [68]. Interestingly, DNA loci covered by QTL5 andQTL24 both carry a gene encoding protochlorophyllide reductase, an enzyme of the chlorophyll biosynthetic pathway [68]. This result suggests that despite their lack of green coloration, leucoembryophyte seeds such as maize grain might produce chlorophylls that, once degraded and recycled, might provide PPP for tocopherol biosynthesis. A biochemical analysis of developing maize grain showed that, whereas they lack any macroscopic green coloration, embryos accumulate very low but detectable traces of the chlorophyll immediate precursor chlorophyllide *a*, of chlorophylls *a* and *b*, and of the first chlorophyll catabolite that lacks the Mg atom pheophytin *a*. In addition, endosperm also accumulated detectable traces of chlorophyll *a*. Although further studies—notably with genetic evidence—are required to definitively link chlorophyll metabolism and tocopherol biosynthesis in leucoembryophyte seeds, these data indicate that leucoembryophyte seeds might produce tocopherols *via* the same mechanism originally described in chloroembryophyte seeds [80,83]. This exiting perspective would definitively link tocopherol metabolism to the one of chlorophylls in plants, and open news questions regarding tocopherol metabolism in non-photosynthetic organisms such as in *Plasmodium falciparum* [14,15,16].

**Figure 4:**
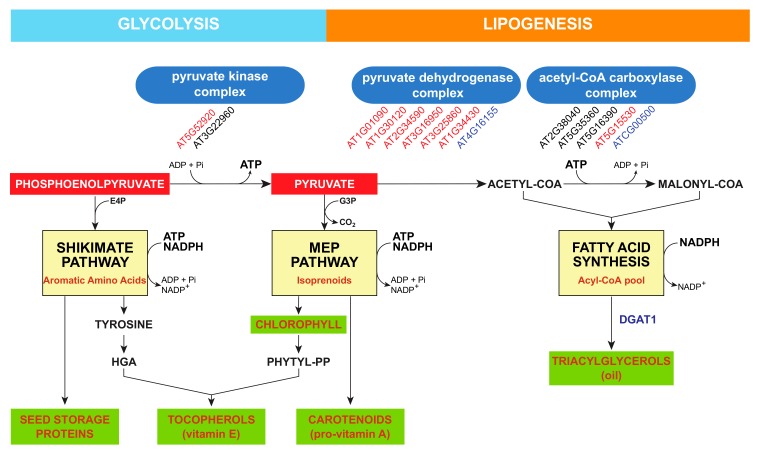
Plastidic intersection of the shikimate pathway, the methyl erythritol phosphate pathway, and lipogenesis. The shikimate pathway, methyl erythritol phosphate (MEP) pathway, and fatty acid synthesis are localized in plastids, and share phosphoenolpyruvate and pyruvate as common biosynthetic precursors. The biosynthesis of triacylglycerols occurs in the endoplasmic reticulum. Arabidopsis Genome Initiative numbers in red indicate that the corresponding gene is downregulated in Arabidopsis *wri1* developing embryos, or upregulated in transgenic plants constitutively expressing the *WRI1* transcription factor. AGI numbers in black indicate that the expression of the corresponding gene is WRI1-independent. AGI numbers in blue indicate that no transcriptomic data was available. Abbreviations: DGAT1, acylCoA:diacylglycerol acyltransferase 1; E4P, erythrose 4-phosphate; G3P, glyceraldehyde 3-phosphate; phytyl-PP, phytyl pyrophosphate.

Although the chlorophyllic origin of tocopherols is clearly established in the seeds and leaves of chloroembryophyte species such as Arabidopsis, little is known about the regulation of PPP biosynthesis linked to tocopherol accumulation. Recently, two novel Arabidopsis ethane methylsulfonate mutants, the *enhanced vitamin e* (*eve*) *1* and *4* mutants, have been isolated by forward genetics [21]. The seeds of *eve1* and *eve4* mutants overaccumulate tocopherols, PC-8, and γ-tocomonoenol, a monounsaturated form of tocochromanols. Interestingly, both *eve1* and *4* seeds did not accumulate any tocotrienols, such as transgenic Arabidopsis plants overaccumulating HGA, suggesting that although HGA metabolism is likely increased in these mutants to sustain tocochromanol metabolism, HGA overaccumulation is not the primary source of its enhancement. In addition, γ-tocomonoenol accumulation has never been reported in Arabidopsis HGA overaccumulating lines, further suggesting that the misregulation of HGA biosynthesis is likely not the primary source of the enhancement of tocochromanol accumulation in *eve1* and *eve4* seeds. The fact that all tocochromanol forms are overaccumulated in *eve1* and *eve4* seeds likely suggests that both mutations affect the chloroplastic isoprenoid metabolism produced by the methyl erythritol phosphate pathway (Figure 4). The mutation responsible for the *eve1* seed phenotype affects the *WRINKLED1* gene that encodes for a transcription factor containing two APETALA2/ethylene response element DNA-binding proteins [84]. This gene is mostly expressed during seed development, although moderate expression has been also detected in roots and flowers [85,86].

Targets of the WRI1 transcription factor in plant genomes have been investigated by transcriptomic studies and in vitro enzymatic assays in developing Arabidopsis *wri1* embryos [85,86,87,88], in the leaves of transgenic maize lines overexpressing the *ZmWRI1a* gene [89], and in *Nicotiana benthamiana* leaves agroinfiltrated with Arabidopsis, potato, poplar, oat, and nutsedge *WRI1* orthologs [90]. The transcription factor WRI1 regulates the expression of several key biosynthetic genes involved in late glycolysis, fatty acid synthesis, and lipid assembly [85,86,87,88,89]. Thus, WRI1 pushes carbon into fatty acid/lipid metabolism by upregulating key lipid biosynthetic genes. It is interesting to note that the genes encoding almost all of the subunits of the plastidial pyruvate dehydrogenase complex are either downregulated in *wri1* developing embryos or upregulated in transgenic plants overexpressing *WRI1* (Figure 4). Interestingly, tocochromanols are synthetized in chloroplasts as well, and the biosynthetic pathways providing both tocochromanol precursors, i.e., the MEP pathway and the shikimate pathway, use the same biosynthetic precursors, i.e., pyruvate and phosphoenolpyruvate, as those used for fatty acid/lipid synthesis (Figure 4). These suggest that tocochromanol and lipid biosyntheses might compete for these biosynthetic precursors.

Another model explaining the enhancement of tocochromanol metabolism in *wri1* seeds might be considered as well. The transient *WRI1* expression performed in *Nicotiana benthamiana* leaves revealed a novel aspect of WRI1 transcriptome that has not been described neither in transgenic lines constitutively expressing *WRI1* nor in *wri1* seeds. Numerous genes encoding chlorophyll biosynthetic enzymes such as protochlorophyllide oxidoreductase and Mg chelatase, chlorophyll-binding proteins, subunits of the light harvesting complexes, and ATP generation complex subunits were strongly downregulated in tobacco leaves transiently expressing *WRI1* constructs (Supplementary file 6 of Grimberg et al. [90]). The authors concluded that the transient expression of *WRI1* is strongly associated with the downregulation of photosynthesis, including energy and reducing equivalent production, respectively. Interestingly, the endogenous *WRI1* gene is not constitutively expressed in Arabidopsis plants; instead, it is transiently expressed in the developing Arabidopsis embryo [85]. This indicates that the *WRI1* expression in the developing Arabidopsis embryo also might downregulate photosynthesis, and thus the production of ATP, as well as reduce equivalents in embryos. Now, the MEP pathway that produces the building blocks for chloroplastic isoprenoids is strongly dependent on ATP and reducing equivalents such as NADPH (Figure 4). Interestingly, it has been suggested that both ATP and NADPH might be key regulatory components of the MEP pathway [91]. This hypothesis suggests that the enhancement of the tocochromanol metabolism in *wri1* seeds might reflect the higher ATP and NADPH production resulting from the reduced repression of seed photosynthesis.

## 6. Conclusions and Perspectives

From the first purification of α-tocopherol from wheat germ oil to the latest model of the tocochromanol biosynthetic pathways introducing tocomonoenol biosynthetic genes, our understanding of vitamin E biosynthesis in plants has been considerably enriched [3,21]. In contrast, much less is known about the mechanisms regulating vitamin E biosynthesis in plants. The fact that tocochromanol biosynthesis mobilizes two distinct biosynthetic pathways, the shikimate pathway and the MEP pathway, requires the degradation of two compounds such as l-tyr and chlorophylls, involves a yet-unknown transport system between the cytoplasm and the stroma in plant species in which HGA biosynthesis is localized in the cytoplasm, and is interconnected to fatty acid biosynthesis in plastids, render the understanding of its regulation particularly challenging. Indeed, understanding the regulation of vitamin E biosynthesis will imply that we take up the challenges to understand the regulation of each of these numerous events. Beyond understanding the regulation of vitamin E biosynthesis in plants, it seems important that medical scientists assess the sanitary consequences of the significant vitamin E deficiency independently detected in human populations, including in developed countries. The fundamental role of this vitamin in human reproduction and its benefit in current widespread diseases such as high cholesterol and neurodegenerative pathologies makes it a candidate of choice to improve human health.
